# Effects of Dietary Nitrate Supplementation on Weightlifting Exercise Performance in Healthy Adults: A Systematic Review

**DOI:** 10.3390/nu12082227

**Published:** 2020-07-26

**Authors:** Alejandro F. San Juan, Raul Dominguez, Ángel Lago-Rodríguez, Juan José Montoya, Rachel Tan, Stephen J. Bailey

**Affiliations:** 1 Department of Health and Human Performance, Sport Biomechanics Laboratory, Facultad de Ciencias de la Actividad Física y del Deporte—INEF, Universidad Politécnica de Madrid, 28040 Madrid, Spain; alejandro.sanjuan@upm.es; 2 Faculty of Health Science, Universidad Isabel I, 09003 Burgos, Spain; angel.lago@ui1.es; 3 Faculty of Medicine, School of Medicine of Physical Education and Sport, Complutense University, 28040 Madrid, Spain; jjmontoya@ucm.es; 4 Faculty of Sports Medicine, Natural Sciences Division, Pepperdine University, Malibu, CA 90263, USA; rachel.tan@pepperdine.edu; 5 School of Sport, Exercise and Health Sciences, Loughborough University, Loughborough, LE11 3TU, UK; s.bailey2@lboro.ac.uk

**Keywords:** beetroot, ergogenic aid, exercise, nutrition, muscle

## Abstract

Dietary nitrate (NO_3_^−^) supplementation has been evidenced to induce an ergogenic effect in endurance and sprint-type exercise, which may be underpinned by enhanced muscle contractility and perfusion, particularly in type II muscle fibers. However, limited data are available to evaluate the ergogenic potential of NO_3_^−^ supplementation during other exercise modalities that mandate type II fiber recruitment, such as weightlifting exercise (i.e., resistance exercise). In this systematic review, we examine the existing evidence basis for NO_3_^−^ supplementation to improve muscular power, velocity of contraction, and muscular endurance during weightlifting exercise in healthy adults. We also discuss the potential mechanistic bases for any positive effects of NO_3_^−^ supplementation on resistance exercise performance. Dialnet, Directory of Open Access Journals, Medline, Pubmed, Scielo, Scopus and SPORT Discus databases were searched for articles using the keywords: nitrate or beetroot and supplement or nut*r or diet and strength or “resistance exercise” or “resistance training” or “muscular power”. Four articles fulfilling the inclusion criteria were identified. Two of the four studies indicated that NO_3_^−^ supplementation could increase aspects of upper body weightlifting exercise (i.e., bench press) performance (increases in mean power/velocity of contraction/number of repetitions to failure), whereas another study observed an increase in the number of repetitions to failure during lower limb weightlifting exercise (i.e., back squat). Although these preliminary observations are encouraging, further research is required for the ergogenic potential of NO_3_^−^ supplementation on weightlifting exercise performance to be determined.

## 1. Introduction

Weightlifting exercise is well established as an exercise modality of resistance exercise to improve skeletal muscle mass [1,2], strength [3,4,5], endurance [6,7] and power [8,9]. These positive adaptations in skeletal muscle function translate into athletic performance [10,11,12,13,14] and health-related [15,16,17,18,19] benefits in a range of populations [20,21,22]. To achieve specific muscular adaptations, resistance exercise training programs can manipulate variables such as muscle action, loading and volume, exercise selection and order, free weights vs. resistance machines, rest periods, number of repetitions and sets, velocity of muscle action and frequency [23]. It is well documented that a propensity for high muscular power production, velocity of contraction and endurance are required for optimal performance in various sports [13,24] and that resistance exercise training can improve these performance determinants [8].

There are different methods to assess muscle strength and power [25]. Static methods include isometric muscle strength assessments to evaluate maximal voluntary isometric contraction (MVIC) force and/or the rate of force development (RFD) at a fixed muscle joint angle. Single limb isokinetic methods allow for the assessment of muscle torque, work and power along a joint’s full range of motion (ROM) (i.e., single knee extension and/or flexion movement). A dynamic method can assess one repetition maximum (1RM) strength and maximum power developed against either a constant (i.e., free weights and exercise machine) or variable (i.e., exercise machine) resistance along a single joint’s full ROM (i.e., bicep curl: elbow joint) or exercises involving multiple-joints (i.e., back squat: ankle, knee and hip joints). Most actions performed in daily physical activities (i.e., walking up and down stairs, handling, press and push) and sports actions (i.e., run, jump, throw) include dynamic muscle contractions, which involve repetitive concentric and eccentric muscle contractions and an associated stretch-shortening cycle (SSC) [26]. However, since isometric methods only assess muscle strength at a fixed joint angle, isokinetic methods measure strength only within a single limb in a specific joint range of motion, and neither of these assessment approaches involve an SSC [27], the application of the findings from such assessments into sporting actions is limited [28,29,30,31,32].

There is also interest in the application of dietary interventions in conjunction with resistance exercise training in an attempt to augment resistance training adaptations and, by extension, sport-specific exercise performance [11,33]. Dietary supplements, such as creatine, caffeine and sodium bicarbonate, have a strong historical evidence basis to support ergogenic effects in certain exercise settings [34]. More recently, inorganic nitrate (NO_3_^−^) ingestion, often administered as concentrated NO_3_^−^-rich beetroot juice (BR), has been reported to confer ergogenic effects in various exercise modalities [35], including running [36,37,38,39,40,41,42,43,44,45,46,47], rowing [48,49], kayaking [50], knee extensions [37,51] and cycling [52,53,54,55,56,57,58,59,60,61,62,63,64]. Although an ergogenic effect of NO_3_^−^ supplementation appears less likely in endurance-trained individuals, i.e., [65,66,67,68,69,70,71,72,73,74], recent systematic reviews support its efficacy as an ergogenic aid during continuous endurance-type exercise [75,76,77] and high-intensity intermittent-type exercise [78].

Dietary NO_3_^−^ supplementation has been observed to elevate nitric oxide (NO) bioavailability via the reduction of exogenous NO_3_^−^ to nitrite (NO_2_^−^) by commensal anaerobic bacteria in the oral cavity [79], followed by the one-electron reduction of NO_2_^−^ to NO (and other nitrogen intermediates) catalyzed by various NO_2_^−^ reductases [80,81,82,83,84] in the tissue and blood. The reduction of NO_2_^−^ to NO is potentiated under conditions of hypoxia [85] and acidosis [86], as are known to occur intramuscularly during exercise [87]. Elevations in [NO_3_^−^] and [NO_2_^−^] following NO_3_^−^ supplementation have been observed in skeletal muscle [88,89,90,91] and plasma [53,64,92], and are associated with positive physiological effects [41,64,74,93] that facilitate a greater capacity for muscular work [51,53,59] and/or improved muscle contractile efficiency (i.e., a lower high-energy phosphate cost of force production) [51,94]. The elevation of plasma [NO_2_^−^] is dependent on methodological considerations, such as the supplementation regimen (i.e., dosage of NO_3_^−^, timing and duration) [64], and there is evidence to suggest that performance enhancement may be more likely after chronic, compared to acute, NO_3_^−^ supplementation [56,63,67].

Although the effects of NO_3_^−^ supplementation on performance during continuous endurance and high-intensity intermittent exercise have been investigated in numerous studies [35], its effects on the contractile properties of isolated muscle groups completing weightlifting exercise has received comparatively limited empirical investigation. There is some evidence that NO_3_^−^ supplementation can enhance force production during voluntary and evoked isometric assessments [73,95,96] and isokinetic voluntary knee extensor power and velocity [97]. Data from animal studies support these observations and have indicated that 7 days of NO_3_^−^ supplementation increased evoked force production in rodents at low-stimulation frequencies and the rate of force development at high-contraction frequencies compared to age-matched controls [98]. These improvements in skeletal muscle contractile function were accompanied by increased protein expression of calcium (Ca^2+^)-handling proteins in the extensor digitorum longus, which is predominantly comprised of type II muscle fibers, but not the soleus, which is predominantly comprised of type I muscle fibers [98]. However, in contrast to the rodent studies, there was an increase in evoked contractile force after NO_3_^−^ supplementation without an effect on muscle Ca^2+^-handling proteins in human skeletal muscle [96]. Collectively, these findings suggest that dietary NO_3_^−^ supplementation has the potential to increase contractile force production, skeletal muscle power and velocity of contraction, particularly in type II muscle fibers, which are heavily recruited during weightlifting exercise [99]. However, findings regarding involuntary contractions evoked by neuromuscular electrostimulation (NMES) may not readily translate to voluntary contractions, since there are some important differences between NMES and voluntary actions [100]. Specifically. motor unit recruitment during NMES is spatially fixed, temporally synchronous and nonselective (i.e., randomized), such that it may not conform to the orderly recruitment of motor units during voluntary contractions [101].

In addition to enhancing force production during single muscle contractions, NO_3_^−^ supplementation has the potential to enhance performance during repeated sub-maximal knee-extensor contractions continued to failure [51]. This increased time to task failure following NO_3_^−^ supplementation was accompanied by lower rates of ATP and PCr turnover, and ADP and Pi accumulation, factors that would be expected to lower skeletal muscle fatigue [102]. In addition, it has been reported that NO_3_^−^ supplementation can lower the PCr cost of muscle force production at the end of a protocol comprising 50 MVIs of the knee extensors [94] and is more effective at improving skeletal muscle contractile function after the muscle has become fatigued [103]. Since resistance exercise training sessions typically comprise a series of sets to task failure using the same exercise modality with a relatively short recovery period, overall performance in a resistance exercise training session will also be influenced by the ability to recover between sets. The recovery of muscle force during repeated bouts of high-intensity exercise is linked to muscle PCr resynthesis [104,105,106], which is largely an O_2_-dependent process [107,108]. Since NO_3_^−^ supplementation has been reported to increase skeletal muscle blood flow, with a preferential shunting of blood flow to type II muscle fibers [109], this has the potential to aid recovery between sets during a resistance exercise training session, which might translate into more repetitions completed in the training session.

Despite the evidence outlined above, which suggests that NO_3_^−^ supplementation has the potential to enhance resistance exercise performance during voluntary isometric and/or isokinetic assessments, and muscle isometric contractions evoked by NMES, a limited number of studies have assessed the potential for ergogenic effects of NO_3_^−^ supplementation on a more transferable form of resistance exercise, such as weightlifting performance. The aim of this review was to provide an up-to-date summary of data from experimental studies that have examined the efficacy of dietary NO_3_^−^ supplementation to improve weightlifting performance (i.e., muscle force production, velocity of contraction, muscular endurance) in healthy adults and to discuss potential physiological mechanisms that may underpin these effects.

## 2. Methodology

**Figure 1:**
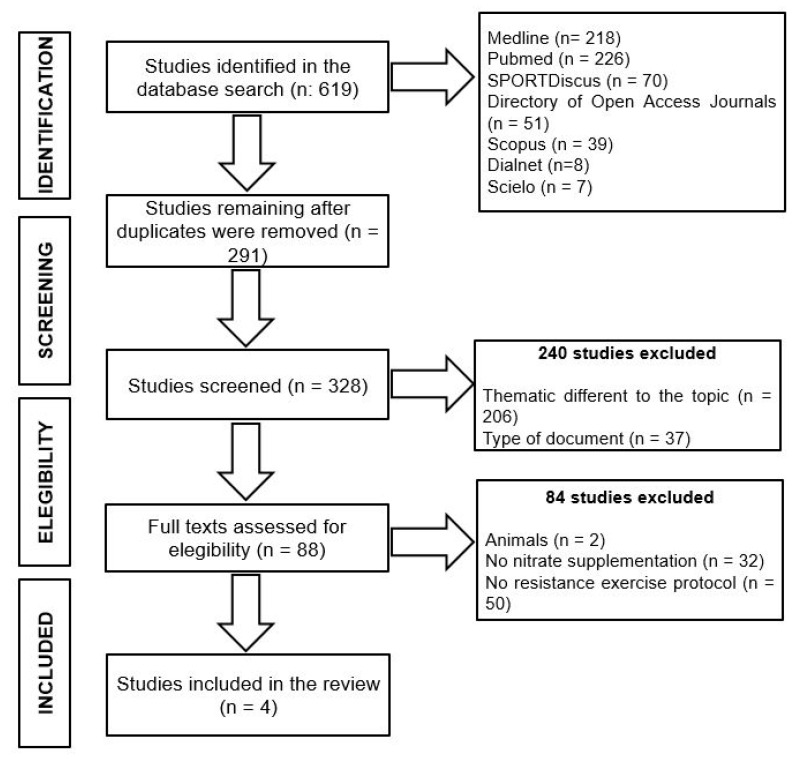
Preferred Reporting Items for Systematic Reviews and Meta-Analysis (PRISMA) flowchart.

A systematic search using the Preferred Reporting Items for Systematic Reviews and Meta-Analysis (PRISMA) guidelines [110] was conducted for studies that investigated NO_3_^−^ supplementation on weightlifting exercise performance using Dialnet, Directory of Open Access Journals, Medline, Pubmed, Scielo, Scopus and SPORTDiscus databases until April 2020, using the following terms: (concept 1) (nitrate OR beet *) AND (concept 2) (supplement * OR nutr * OR diet *) AND (concept 3) (strength OR “resistance exercise” OR “resistance training” OR “muscular power”). The original search yielded a total of 619 studies. After the elimination of duplicate articles and screening for inclusion criteria, a total of 291 articles were independently read and reviewed by three authors (RD, JJM and ASF). A quality assessment procedure was performed by three authors (ALR, RD and JJM) using the PEDro scale [111]. A total of four articles met the eligibility criteria for the present systematic review (Figure 1).

To ensure that the selection of studies assessed the effects of NO_3_^−^ supplementation on weightlifting exercise performance, the authors applied a set of inclusion criteria [112]:

1. Studies that were published as a full article (i.e., not a conference abstract) and performed in healthy humans (aged 18 to 65 years).
2. Studies that included a NO_3_^−^ and a placebo intervention.
3. Studies which assessed voluntary dynamic resistance strength (i.e., not isometric or isokinetic strength and not involuntary muscle contractions evoked by NMES).
4. Studies that included any of the following variables: i) one repetition maximum (1RM); ii) power or velocity movement; iii) number of repetitions to failure with submaximal loads.

The four studies selected for our systematic review included a total of 49 men, all of whom were resistance trained (i.e., performed resistance exercise a minimum of twice per week).

In two of the selected studies [113,114], the influence of acute BR ingestion was assessed by adminstering 1 × 70 mL of BR (~6.4 mmol of NO_3_^−^ per 70 mL) ~2 h prior to the commencement of exercise. In the remaining two studies [115,116], longer-term (≥ 3 days) dosing strategies of NO_3_^−^ supplementation were employed. Mosher et al. [116] administered 1 × 70 mL of BR per day (~6.4 mmol of NO_3_^−^ per 70 mL) for 6 consecutive days, although the authors did not report the timing of ingestion, which has important implications for the elevation of plasma NO_3_^−^ and NO_2_^−^ [64]. Flanagan et al. [115] administered 2 × NO_3_^−^-rich performance bars (32.5 mg of NO_3_^−^ per two bars) for 3 consecutive days with the final two NO_3_^−^-rich performance bars ingested ~60 min prior to the commencement of exercise.

## 3. Results and Discussion

The exercise modalities used to assess weightlifting exercise performance were bench press using free weights [113], bench press using a Smith machine [114,116] and box squats using a Smith machine [114,115]. The details of the performance tests employed are summarized in Table 1.

This is the first systematic review to have focused on the ergogenic effect of dietary NO_3_^−^ supplementation on weightlifting exercise performance. The main findings were that dietary NO_3_^−^ supplementation can increase muscular power and velocity, and the number of repetitions to failure during bench press exercise, but not box squat exercise, in resistance-trained males.

### 3.1. The Effects of Dietary Nitrate Supplementation on Weightlifting Exercise Performance

Williams et al. [113] examined the effect of acute dietary NO_3_^−^ supplementation (BR ingested 2 h prior to exercise) on muscle power, velocity and number of repetitions to failure during free-weight bench press exercise at 70%1RM in resistance-trained men. The authors observed a 19.5% increase in mean power, a 6.5% increase in mean velocity, and a 10.7% increase in the number of repetitions to failure [113]. In another study, Ranchal-Sánchez et al. [114] observed an enhancement in the number of repetitions to failure (+17.7%) in the sum of sets for bench press and back squat with loads of 60%, 70% and 80% 1RM after NO_3_^−^ supplementation (BR ingested 2 h prior to exercise), although authors failed to find an effect on muscular velocity and power. These conflicting findings may be attributed to inter-study differences in the protocols used to assess muscular power and velocity. Indeed, whereas Williams et al. [113] assessed muscle power and velocity during two single explosive repetitions with full recovery (5 min rest between sets), Ranchal-Sánchez et al. [114] assessed power and velocity during sets of repetitions until failure. Muscle velocity and muscle power assessment require optimal neuromuscular conditions and, as such, studies analyzing the effect of different supplements on muscular velocity and power selected a maximum of two repetitions with a submaximal load, with recovery periods of 2−5 min [117,118,119,120,121,122]. Thus, the procedure used by Ranchal-Sánchez et al. [114] to assess muscle power and velocity may not be suitable to detect a potential effect of NO_3_^−^ supplementation. Longer-term NO_3_^−^ supplementation was also observed to be effective, as 6 days of BR supplementation increased the number of repetitions to failure (+19.4%) and increased the total amount of weight lifted (+18.9%) during Smith machine bench press exercise at 60%1RM in resistance-trained men [116]. Therefore, the existing evidence suggests that acute and short-term NO_3_^−^ supplementation can improve bench press performance in resistance-trained males. In contrast, Flanagan et al. [115] did not observe any change in the number of repetitions to failure during box squat exercise at 60%1RM in resistance-trained men following the administration of NO_3_^−^-rich performance bars over 3 days. A limitation in Flanagan et al. [115] was the low NO_3_^−^ dose administered. Specifically, Flanagan et al. [115] administered 32.5 mg (~0.5 mmol) of NO_3_^−^ daily, which is markedly lower than Williams et al. [113] (6.4 mmol NO_3_^−^ acutely) and Mosher et al. [116] (6 days of 6.4 mmol NO_3_^−^ daily), both of whom observed improved resistance exercise performance. Since plasma [NO_2_^−^] increases dose-dependently after NO_3_^−^ supplementation and is correlated with enhanced exercise capacity [64], the low NO_3_^−^ dose administered in the study of Flanagan et al. [115] is likely to have underpinned the lack of effect of NO_3_^−^ supplementation in that study. This interpretation is reinforced by Coggan et al. [123] who reported that the relative magnitude of the increase in knee-extensor peak power output following NO_3_^−^ ingestion was positively correlated with the increase in plasma [NO_2_^−^]. However, a limitation of all existing studies assessing the effect of NO_3_^−^ supplementation on resistance exercise performance is the lack of plasma [NO_2_^−^] determination.

In addition to inter-study differences in the dosing strategies, the exercise modality (upper body vs. lower body) employed might also have contributed to the disparate findings across studies assessing the ergogenic potential of NO_3_^−^ supplementation on resistance exercise performance to date. Indeed, two studies reported improved resistance exercise performance after NO_3_^−^ supplementation during bench press exercise [113,116], whereas squat performance was not improved after NO_3_^−^ supplementation in the study by Flanagan et al. [115], but the total number of repetitions during three sets of back squats was enhanced in the study by Ranchal-Sánchez et al. [114]. Given that there is evidence to suggest that NO_3_^−^ supplementation may be more effective at enhancing physiological responses in type II muscle fibers [124] and since the proportion of type II muscle fibers may be greater in the upper body musculature, i.e., [125], this might account for the improved bench press and the inconsistent effects observed on squat performance after NO_3_^−^ supplementation. However, there is evidence that weightlifting training increases both the hypertrophy and proportion of type II muscle fibers, such that the proportion of type II muscle is greater in resistance-trained individuals [126,127]. Accordingly, this could partly account for the improvements observed in Mosher et al. [116], Williams et al. [113] and Ranchal-Sánchez et al. [114], who recruited resistance-trained men.

Taken together, the existing, albeit limited, evidence suggests that acute and short-term dietary NO_3_^−^ supplementation can enhance weightlifting exercise performance by increasing muscle power production, velocity of contraction and muscular endurance in healthy resistance-trained adults. However, the results are incongruous with inconsistencies likely linked to differences in supplementation strategies and exercise modality. Therefore, further research is required to assess the weightlifting exercise settings and populations in which NO_3_^−^ supplementation is more or less likely to be ergogenic. Moreover, while encouraging preliminary evidence suggests that dietary NO_3_^−^ supplementation may enhance weightlifting training quality, further research is also required to assess whether this translates into greater adaptations to chronic resistance exercise training.

### 3.2. Physiological Mechanisms

Consistent with the potential for improved weightlifting exercise performance after NO_3_^−^ supplementation, enhanced skeletal muscle contractile function has been observed during electrically stimulated contractions [95,96,103], and enhanced peak power output has been observed during isokinetic dynamometry [97,123,128] and cycling [45,57,60,129,130,131,132,133] exercise. Although the exact physiological mechanisms responsible for enhanced exercise performance following dietary NO_3_^−^ supplementation are unclear, a number of putative mechanisms have been identified which could contribute to improved weightlifting exercise performance.

Using a mouse model, Hernández et al. [98] demonstrated that 7 days of NO_3_^−^ supplementation increased the rate of force development at 100 Hz by 35% and force production at 50 Hz during evoked skeletal muscle contractions at a supraphysiological PO_2_. The increase in evoked force production was accompanied by the increased expression of Ca^2+^-handling proteins, dihydropyridine receptors (DHPRs) and calsequestrin (CASQ) in type II but not type I skeletal muscle [98]. There is also previous evidence indicating that NaNO_2_ administration can increase cytosolic [Ca^2+^] without altering force production at a supraphysiological PO_2_ [134], or lower cytosolic [Ca^2+^] concomitant with lower submaximal, but not maximal, force at a physiological PO_2_ [118], during single evoked isometric contractions in isolated mouse muscle fibers. However, during a repeated, fatigue-inducing contraction protocol, NaNO_2_ administration increased time to task failure by offsetting the reductions in Ca^2+^ pumping rate and Ca^2+^ sensitivity [135]. While these data suggest that increasing the exposure of mouse skeletal muscle to NO_3_^−^ and/or NO_2_^−^ can modulate skeletal muscle contractility via changes in skeletal muscle Ca^2+^ handling, the findings from Whitfield et al. [96] challenge the notion that improved skeletal muscle contractile function after NO_3_^−^ supplementation in human skeletal muscle is linked to increased content of Ca^2+^-handling proteins. Specifically, these authors observed an increased force production and rate of force production during evoked isometric twitches in healthy humans without changes in skeletal muscle CASQ, DHPR or SERCA protein content following 7 days of BR supplementation.

Another mechanism that could improve skeletal muscle contractile function after NO_3_^−^ supplementation is the post-translational modification of the skeletal muscle contractile or Ca^2+^-handling proteins [136]. Indeed, NO can react with protein thiols (i.e., moieties containing sulfhydryl groups, RSH or RS^−^) to form RSNO groups in a reversible process termed S-nitrosylation [137]. S-nitrosylation and denitrosylation alter the structural conformation and thus function of proteins [138]. For example, NO has been reported to S-nitrosylate myosin heavy chains in skeletal muscle, leading to increased contractile force [139]. The potential influence of S-nitrosylation on excitation–contraction coupling is complex given that various contractile-related proteins can undergo reversible post-translation modifications at cysteine residues on thiols, such as myosin [140], troponin [141], SERCA [142] and ryanodine receptors (RyRs) [143,144], and that these post-translation protein modifications are likely dependent on interactions between NO, reactive oxygen species and glutathione bioavailability [145]. In addition, RyR proteins contain a markedly greater number of sulfhydryl groups compared to other contractile proteins [146], which supports the proposed hypothesis that NO-mediated RyR modulation and Ca^2+^ release could contribute to enhanced muscle contractility following NO_3_^−^ supplementation [123]. Importantly, these effects could occur independent of changes in the content of Ca^2+^-handling proteins. An interesting observation by Flanagan et al. [115] was that EMG amplitude increased during weightlifting exercise after NO_3_^−^ supplementation despite no change in weightlifting exercise performance. However, other studies have not observed changes in EMG after NO_3_^−^ supplementation [95,103] and, as such, it is unclear whether NO_3_^−^ supplementation alters neural drive. Further research is required to evaluate how NO_3_^−^ supplementation can modulate excitation–contraction coupling in human skeletal muscle.

In addition to potential changes to excitation–contraction coupling proteins, NO_3_^−^ supplementation has been reported to alter high-energy phosphate turnover and phosphorus metabolites in human skeletal muscle [51,94]. Specifically, NO_3_^−^ supplementation has been reported to lower the high-energy phosphate cost of skeletal muscle contractile force production [51,94] and the intramuscular accumulation of ADP and Pi [51], factors which would be expected to abate the development of skeletal muscle fatigue [102]. Dietary NO_3_^−^ supplementation has also been shown to increase muscle blood flow [109], which might aid muscle PCr resynthesis between sets to failure [107,108] and the recovery of force and performance [104,105,106].

Taken together, the existing evidence suggests that NO_3_^−^ supplementation can improve skeletal muscle contractile function and might enhance weightlifting exercise performance in humans. Therefore, NO_3_^−^ supplementation holds promise as an effective nutritional ergogenic aid for weightlifting exercise. The potential candidate mechanisms for improved weightlifting exercise performance after NO_3_^−^ supplementation include enhanced excitation–contraction coupling, via modulation of Ca^2+^-handling and contractile proteins [98,134,135,139]; improved skeletal muscle metabolic control, via lowering the high-energy phosphate cost of contraction and fatigue-related metabolite accumulation [51,94]; and improved skeletal muscle perfusion [109]. However, further research is required to resolve the mechanisms for improved weightlifting exercise performance after NO_3_^−^ supplementation. Furthermore, while NO_3_^−^ supplementation appears to potentially enhance resistance training quality, it is unclear if this will translate into improved weightlifting training adaptations. Notably, although NO_3_^−^ supplementation has been reported to enhance the adaptations to sprint interval training [62,147], the molecular bases for skeletal muscle oxidative metabolism and hypertrophy training adaptations are different and can be potentially antagonistic [148,149]. For example, NO_2_^−^ has been reported to activate AMPK [150], which is a key regulator of skeletal muscle oxidative metabolism adaptations, but interferes with mTORC1 signaling, which is a master regulator of skeletal muscle hypertrophy [148,149]. Therefore, further research is required to assess how NO_3_^−^ supplementation impacts chronic adaptations to weightlifting exercise training.

## 4. Limitations

Although there are numerous studies analyzing the effect of NO_3_^−^ on various aspects of exercise performance, the number of high-quality studies (i.e., randomized controlled trials) focused on weightlifting exercise is limited, which restricted the sample analyzed in the present systematic review. In addition, existing between-study differences in the supplementation dosage (from 32.5 mg NO_3_^−^ to 6.4 mmol NO_3_^−^) and the period of supplementation (from acute to chronic over 6 days), along with differences regarding the type of exercise selected, prevented a firm conclusion on the ergogenic potential of NO_3_^−^ supplementation on weightlifting exercise performance at this stage. Nevertheless, this systematic review is an important contribution to the literature as it highlights both the potential promise of NO_3_^−^ supplementation as an ergogenc aid for weightlifting exercise performance and the necessity to conduct further studies to improve understanding on this topic.

## 5. Conclusions

In conclusion, the limited exisiting literature suggests that acute and short-term dietary NO_3_^−^ supplementation holds promise as a nutritional intervention to enhance weightlifting performance in resistance-trained males. Indeed, NO_3_^−^ supplementation can improve muscular power production, velocity of contraction, and the number of repetitions to failure during weightlifting exercise. Given the important athletic and clinical implications of improved weightlifting exercise performance, NO_3_^−^ supplementation might offer potential as an ergogenic and therapeutic nutritional aid. The mechanistic bases responsible for the potential ergogenic effect of NO_3_^−^ supplementation on weightlifting exercise performance may be linked to improvements in skeletal muscle excitation–contraction coupling, high-energy phosphate metabalism and perfusion. However, further research is required to resolve the putative underlying mechanisms for, and the conditions in which, NO_3_^−^ supplementation might enhance weightlifting exercise performance, as well as its effects on chronic adaptations to weightlifting exercise training.

## Figures and Tables

**Table 1:** Studies assessing the effects of dietary NO_3_^−^ supplementation on resistance exercise performance in humans.

Reference	Subjects	Supplementation	Exercise Protocol	Findings
Flanagan et al. (2016) [115]	Fourteen resistance-trained men	Three days and 60 min prior to exercise ingestion of 2 × NO_3_^−^-rich bars (32.5 mg NO_3_^−^·d^−1^)	Smith machine box squats: three sets x 3-s isometric squats interspersed with 120-s rest, then dynamic box squats @ 60%1RM with 10% increases up to 90%1RM, then 10% decreases to 60%1RM, then RTF on last 60%1RM set	↔ RTF: −1.5% (599 ± 5 vs. 608 ± 5 reps) ↑ EMG amplitude: +5% (83 ± 3 vs. 79 ± 4%)
Mosher et al. (2016) [116]	Twelve resistance-trained men	Six days of 1 × 70 mL NO_3_^−^ rich BR supplementation (~6.4 mmol NO_3_^−^·d^−1^)	Smith machine bench press: three sets of RTF @ 60%1RM interspersed with 2 min of recovery between sets	↑ RTF: +19.4% ↑ total weight lifted: +18.9% (2583 ± 864 vs. 2172 ± 721 kg)
Williams et al. (2020) [113]	Eleven resistance-trained men	Two hours prior to exercise ingestion of 1 × 70 mL NO_3_^−^ rich BR (~6.4 mmol NO_3_^−^)	Free-weight bench press: two sets x 2 explosive reps, 5 min rest, then three sets x RTF @ 70%1RM interspersed with 2 min of recovery between sets	↑ RTF: +10.7% (31 ± 6 vs. 28 ± 6 reps) ↑ *P*_mean_: +19.5% (607 ± 112 vs. 508 ± 118 W) ↑ *V*_mean_: +6.5% (0.66 ± 0.08 vs. 0.62 ± 0.08 m·s^−1^)
Ranchal-Sanchez et al. (2020) [114]	Twelve resistance-trained men	Two hours prior to exercise ingestion of 1 × 70 mL NO_3_^−^ rich BR (~6.4 mmol NO_3_^−^)	Smith machine bench press and back squat: three sets x RTF @ 60−70−80%1RM with 2 min of recovery between sets. After the eccentric phase of each rep, participants rested for 1.0−1.5 s	↑ RTF back squat: +23.4% (60 ± 20 vs. 46 ± 16 reps) ↑ RTF total (sum bench press and back squat): +17.7% (89 ± 25 vs. 75 ± 21 reps)

↑ = significant increase; ↔ = no change; 1RM = one-repetition maximum; BR = beetroot juice; EMG = surface electromyography; m·s^−1^ = meters per second; min = minutes; NO_3_^−^ = nitrate; *P*_mean_ = mean power of bench press; reps = repetitions; RTF = repetitions to failure; s = seconds; *V*_mean_ = mean velocity of bench press; W = Watts.

## References

[1] Joanisse S., Lim C., McKendry J., Mcleod J.C., Stokes T., Phillips S.M. (2020). Recent advances in understanding resistance training-induced skeletal muscle hypertrophy in humans. F1000Research.

[2] Phillips S.M. (2014). A brief review of critical processes in exercise-induced muscular hypertrophy. Sports Med..

[3] Grgic J., Schoenfeld B.J., Davies T.B., Lazinica B., Krieger J.W., Pedisic Z. (2018). Effect of Resistance Training Frequency on Gains in Muscular Strength: A Systematic Review and Meta-Analysis. Sports Med..

[4] Schoenfeld B.J., Ogborn D., Krieger J.W. (2015). Effect of repetition duration during resistance training on muscle hypertrophy: A systematic review and meta-analysis. Sports Med..

[5] Schoenfeld B.J., Ogborn D., Krieger J.W. (2016). Effects of Resistance Training Frequency on Measures of Muscle Hypertrophy: A Systematic Review and Meta-Analysis. Sports Med..

[6] Kell R.T., Bell G., Quinney A. (2001). Musculoskeletal fitness, health outcomes and quality of life. Sports Med..

[7] Tanaka H., Swensen T. (1998). Impact of resistance training on endurance performance. A new form of cross-training?. Sports Med..

[8] Baker D., Nance S., Moore M. (2001). The load that maximizes the average mechanical power output during explosive bench press throws in highly trained athletes. J. Strength Cond. Res..

[9] Cronin J.B., Sleivert G. (2005). Challenges in understanding the influence of maximal power training on improving athletic performance. Sports Med..

[10] Alcaraz-Ibañez M., Rodríguez-Pérez M. (2018). Effects of resistance training on performance in previously trained endurance runners: A systematic review. J. Sports Sci..

[11] Blagrove R.C., Howatson G., Hayes P.R. (2018). Effects of Strength Training on the Physiological Determinants of Middle- and Long-Distance Running Performance: A Systematic Review. Sports Med..

[12] Bolger R., Lyons M., Harrison A.J., Kenny I.C. (2015). Sprinting performance and resistance-based training interventions: A systematic review. J. Strength Cond. Res..

[13] Muniz-Pardos B., Gomez-Bruton A., Matute-Llorente A., Gonzalez-Aguero A., Gomez-Cabello A., Gonzalo-Skok O., Casajus J.A., Vicente-Rodriguez G. (2019). Swim-Specific Resistance Training: A Systematic Review. J. Strength Cond. Res..

[14] Thiele D., Prieske O., Chaabene H., Granacher U. (2020). Effect of strength training on physical fitness and sport-specific performance in recreational, sub-elite, and elite rowers: A systematic review with meta-analysis. J. Sports Sci..

[15] Lauersen J.B., Andersen T.E., Andersen L.B. (2018). Strength training as superior, dose-dependent and safe prevention of acute and overuse sports injuries: A systematic review, qualitative analysis and meta-analysis. Br. J. Sports Med..

[16] Lopez P., Pinto R.S., Radaelli R., Rech A., Grazioli R., Izquierdo M., Cardore E.L. (2018). Benefits of resistance training in physically frail elderly: A systematic review. Aging Clin. Exp. Res..

[17] McLeod J.C., Stokes T., Phillips S.M. (2019). Resistance exercise training as a primary countermeasure to age-related chronic disease. Front. Physiol..

[18] Nakano J., Hashizume K., Fukushima T., Ueno K., Matsuura E., Ikio Y., Ishii S., Morishita S., Tanaka K., Kusuba Y. (2018). Effects of Aerobic and Resistance Exercises on Physical Symptoms in Cancer Patients: A Meta-analysis. Integr. Cancer Ther..

[19] Nery C., Moraes S.R.A., Novaes K.A., Bezerra M.A., Silveira P.V.C., Lemos A. (2017). Effectiveness of resistance exercise compared to aerobic exercise without insulin therapy in patients with type 2 diabetes mellitus: A meta-analysis. Braz. J. Phys. Ther..

[20] Borde R., Hortobágyi T., Granacher U. (2015). Dose-Response Relationships of Resistance Training in Healthy Old Adults: A Systematic Review and Meta-Analysis. Sports Med..

[21] Peitz M., Behringer M., Granacher U. (2018). A systematic review on the effects of resistance and plyometric training on physical fitness in youth- What do comparative studies tell us?. PLoS ONE.

[22] Schoenfeld B.J. (2010). The mechanisms of muscle hypertrophy and their application to resistance training. J. Strength Cond. Res..

[23] American College of Sports Medicine (2009). American College of Sports Medicine Position Stand. Progression Models in Resistance Training for Healthy Adults. Med. Sci. Sports Exerc..

[24] Pareja-Blanco F., Rodríguez-Rosell D., Sánchez-Medina L., Gorostiaga E.M., González-Badillo J.J. (2014). Effect of movement velocity during resistance training on neuromuscular performance. Int. J. Sports Med..

[25] Jaric S. (2002). Muscle strength testing: Use of normalisation for body size. Sports Med..

[26] Tallis J., Duncan M.J., James R.S. (2015). What can isolated skeletal muscle experiments tell us about the effects of caffeine on exercise performance?. Br. J. Pharmacol..

[27] Perrin D.H. (1993). Isokinetic Exercise and Assessment.

[28] Baker D., Wilson G., Carlyon B. (1994). Generality versus specificity: A comparison of dynamic and isometric measures of strength and speed-strength. Eur. J. Appl. Physiol. Occup. Physiol..

[29] Gentil P., Del Vecchio F.B., Paoli A., Schoenfeld B.J., Bottaro M. (2017). Isokinetic Dynamometry and 1RM Tests Produce Conflicting Results for Assessing Alterations in Muscle Strength. J. Hum. Kinet..

[30] Izquierdo M., Häkkinen K., Gonzalez-Badillo J.J., Ibáñez J., Gorostiaga E.M. (2002). Effects of long-term training specificity on maximal strength and power of the upper and lower extremities in athletes from different sports. Eur. J. Appl. Physiol..

[31] González-Badillo J.J., Sánchez-Medina L. (2010). Movement velocity as a measure of loading intensity in resistance training. Int. J. Sports Med..

[32] González-Badillo J.J., Marques M.C., Sánchez-Medina L. (2011). The importance of movement velocity as a measure to control resistance training intensity. J. Hum. Kinet..

[33] Pareja-Blanco F., Rodríguez-Rosell D., Sánchez-Medina L., Sanchis-Moysi J., Dorado C., Mora-Custodio R., Yáñez-García J.M., Morales-Alamo D., Pérez-Suárez I., Calbet J.A.L. (2017). Effects of velocity loss during resistance training on athletic performance, strength gains and muscle adaptations. Scand. J. Med. Sci. Sports.

[34] Maughan R.J., Burke L.M., Dvorak J., Larson-Meyer D.E., Peeling P., Phillips S.M., Rawson E.S., Walsh N.P., Garthe I., Geyer H. (2018). IOC consensus statement: Dietary supplements and the high-performance athlete. Int. J. Sport Nutr. Exerc. Metab..

[35] Jones A.M., Thompson C., Wylie L.J., Vanhatalo A. (2018). Dietary nitrate and physical performance. Annu. Rev. Nutr..

[36] Balsalobre-Fernández C., Romero-Moraleda B., Cupeiro R., Peinado A.B., Butragueño J., Benito P.J. (2018). The effects of beetroot supplementation on exercise economy, rating of perceived exertion and running mechanics in elite distance runners: A double-blinded, randomized study. PLoS ONE.

[37] Lansley K.E., Winyard P.G., Fulford J., Vanhatalo A., Bailey S.J., Blackwell J.R., DiMenna F.J., Gilchrist M., Benjamin N., Jones A.M. (2011). Dietary nitrate supplementation reduces the O2 cost of walking and running: A place-controlled study. J. Appl. Physiol..

[38] Murphy M., Eliot K., Heuertz R.M., Weiss E. (2012). Whole beetroot consumption acutely improves running performance. J. Acad Nutr Diet..

[39] Nyakayiru J., Jonvik K.L., Pinckaers P.J., Senden J., van Loon L.J.C., Verdijk L.B. (2017). Beetroot juice supplementation improves high-intensity intermittent type exercise performance in trained soccer players. Int. J. Sport Nutr. Exerc. Metab..

[40] Peacock O., Tjønna A.E., James P., Wisløff U., Welde B., Böhlke N., Smith A., Stokes K., Cook C., Sandbakk O. (2012). Dietary nitrate does not enhance running performance in elite cross-country skiers. Med. Sci. Sports Exerc..

[41] Porcelli S., Ramaglia M., Bellistri G., Pavei G., Pugliese L., Montorsi M., Rasica L., Mazorati M. (2015). Aerobic fitness affects the exercise performance responses to nitrate supplementation. Med. Sci. Sports Exerc..

[42] Porcelli S., Pugliese L., Rejc E., Pavei G., Bonato M., Montorsi M., La Torre A., Rasica L., Marzorati M. (2016). Effects of a short-term high-nitrate diet on exercise performance. Nutrients.

[43] Sandbakk S.B., Sandbakk Ø., Peacock O., James P., Welde B., Stokes K., Böhlke N., Tjønna A.E. (2015). Effects of acute supplementation of L-arginine and nitrate on endurance and sprint performance in elite athletes. Nitric Oxide.

[44] Shannon O.M., Duckworth L., Barlow M.J., Woods D., Lara J., Siervo M., O’Hara J.P. (2016). Dietary nitrate supplementation enhances high-intensity running performance in moderate normobaric hypoxia, independent of aerobic fitness. Nitric Oxide.

[45] Thompson C., Wylie L.J., Fulford J., Kelly J., Black M.I., McDonagh S.T., Jeukendrup A.E., Vanhatalo A., Jones A.M. (2015). Dietary nitrate improves sprint performance and cognitive function during prolonged intermittent exercise. Eur. J. Appl. Physiol..

[46] Thompson C., Vanhatalo A., Jell H., Fulford J., Carter J., Nyman L., Bailey S.J., Jones A.M. (2016). Dietary nitrate supplementation improves sprint and high-intensity intermittent running performance. Nitric Oxide.

[47] Wylie L.J., Mohr M., Krustrup P., Jackman S.R., Ermidis G., Kelly J., Black M.I., Bailey S.J., Vanhatalo A., Jones A.M. (2013). Dietary nitrate supplementation improves team sport-specific intense intermittent exercise performance. Eur. J. Appl. Physiol..

[48] Bond H., Morton L., Braakhuis A.J. (2012). Dietary nitrate supplementation improves rowing performance in well-trained rowers. Int. J. Sport Nutr. Exerc. Metab..

[49] Hoon M.W., Jones A.M., Johnson N.A., Blackwell J.R., Broad E.M., Lundy B., Rice A.J., Burke L.M. (2014). The effect of variable doses of inorganic nitrate-rich beetroot juice on simulated 2000-m rowing performance in trained athletes. Int. J. Sports Physiol. Perform..

[50] Peeling P., Cox G.R., Bullock N., Burke L.M. (2015). Beetroot Juice Improves On-Water 500 M Time-Trial Performance, and Laboratory-Based Paddling Economy in National and International-Level Kayak Athletes. Int. J. Sport Nutr. Exerc. Metab..

[51] Bailey S.J., Fulford J., Vanhatalo A., Winyard P.G., Blackwell J.R., DiMenna F.J., Wilkerson D.P., Benjamin N., Jones A.M. (2010). Dietary nitrate supplementation enhances muscle contractile efficiency during knee-extensor exercise in humans. J. Appl. Physiol..

[52] Aucouturier J., Boissière J., Pawlak-Chaouch M., Cuvelier G., Gamelin F.-X. (2015). Effect of dietary nitrate supplementation on tolerance to supramaximal intensity intermittent exercise. Nitric Oxide.

[53] Bailey S.J., Winyard P.G., Vanhatalo A., Blackwell J.R., DiMenna F.J., Wilkerson D.P., Tarr J., Benjamin N., Jones A.M. (2009). Dietary nitrate supplementation reduces the O2cost of low-intensity exercise and enhances tolerance to high-intensity exercise in humans. J. Appl. Physiol..

[54] Bailey S.J., Varnham R.L., DiMenna F.J., Breese B.C., Wylie L.J., Jones A.M. (2015). Inorganic nitrate supplementation improves muscle oxygenation, O2 uptake kinetics, and exercise tolerance at high but not low pedal rates. J. Appl. Physiol..

[55] Breese B.C., McNarry M.A., Marwood S., Blackwell J.R., Bailey S.J., Jones A.M. (2013). Beetroot juice supplementation speeds O2 uptake kinetics and improves exercise tolerance during severe-intensity exercise initiated from an elevated metabolic rate. Am. J. Physiol. Integr. Comp. Physiol..

[56] Cermak N.M., Gibala M.J., Van Loon L. (2012). Nitrate supplementation’s improvement of 10-km time-trial performance in trained cyclists. Int. J. Sport Nutr. Exerc. Metab..

[57] Jonvik K., Nyakayiru J., Van Dijk J.-W., Maase K., Ballak S., Senden J., Van Loon L.J.C., Verdijk L.B. (2018). Repeated-sprint performance and plasma responses following beetroot juice supplementation do not differ between recreational, competitive and elite sprint athletes. Eur. J. Sport Sci..

[58] Kelly J., Vanhatalo A., Wilkerson D.P., Wylie L., Jones A.M. (2013). Effects of Nitrate on the Power–Duration Relationship for Severe-Intensity Exercise. Med. Sci. Sports Exerc..

[59] Lansley K.E., Winyard P., Bailey S.J., Vanhatalo A., Wilkerson D.P., Blackwell J.R., Gilchrist M., Benjamin N., Jones A.M. (2011). Acute Dietary Nitrate Supplementation Improves Cycling Time Trial Performance. Med. Sci. Sports Exerc..

[60] Rimer E.G., Peterson L.R., Coggan A.R., Martin J.C. (2016). Increase in Maximal Cycling Power with Acute Dietary Nitrate Supplementation. Int. J. Sports Physiol. Perform..

[61] Rokkedal-Lausch T., Franch J., Poulsen M.K., Thomsen L.P., Weitzberg I., Kamavuako E.N., Karbing D.S., Larsen R.G. (2019). Chronic high-dose beetroot juice supplementation improves time trial performance of well-trained cyclists in normoxia and hypoxia. Nitric Oxide.

[62] Thompson C., Wylie L.J., Blackwell J.R., Fulford J., Black M.I., Kelly J., McDonagh S.T.J., Carter J., Bailey S.J., Vanhatalo A. (2016). Influence of dietary nitrate supplementation on physiological and muscle metabolic adaptations to sprint interval training. J. Appl. Physiol..

[63] Vanhatalo A., Bailey S.J., Blackwell J.R., DiMenna F.J., Pavey T., Wilkerson D.P., Benjamin N., Winyard P.G., Jones A.M. (2010). Acute and chronic effects of dietary nitrate supplementation on blood pressure and the physiological responses to moderate-intensity and incremental exercise. Am. J. Physiol. Integr. Comp. Physiol..

[64] Wylie L.J., Kelly J., Bailey S.J., Blackwell J.R., Skiba P.F., Winyard P.G., Jeukendrup A.E., Vanhatalo A., Jones A.M. (2013). Beetroot juice and exercise: Pharmacodynamic and dose-response relationships. J. Appl. Physiol..

[65] Boorsma R.K., Whitfield J., Spriet L.L. (2014). Beetroot juice supplementation does not improve performance of elite 1500-m runners. Med. Sci. Sports Exerc..

[66] Callahan M.J., Parr E.B., Hawley J.A., Burke L.M. (2017). Single and Combined Effects of Beetroot Crystals and Sodium Bicarbonate on 4-km Cycling Time Trial Performance. Int. J. Sport Nutr. Exerc. Metab..

[67] Cermak N.M., Res P., Stinkens R., Lundberg J.O., Gibala M.J., Van Loon L. (2012). No improvement in endurance performance after a single dose of beetroot juice. Int. J. Sport Nutr. Exerc. Metab..

[68] De Castro T.F., Manoel F.D.A., Machado F. (2018). Beetroot juice supplementation does not modify the 3-km running performance in untrained women. Sci. Sports.

[69] Lowings S., Shannon O.M., Deighton K., Matu J., Barlow M.J. (2017). Effect of Dietary Nitrate Supplementation on Swimming Performance in Trained Swimmers. Int. J. Sport Nutr. Exerc. Metab..

[70] McQuillan J.A., Dulson D.K., Laursen P.B., Kilding A.E. (2017). Dietary Nitrate Fails to Improve 1 and 4 km Cycling Performance in Highly Trained Cyclists. Int. J. Sport Nutr. Exerc. Metab..

[71] Mosher S.L., Gough L.A., Deb S., Saunders B., McNaughton L.R., Brown D.R., Sparks S. (2019). High dose Nitrate ingestion does not improve 40 km cycling time trial performance in trained cyclists. Res. Sports Med..

[72] Oskarsson J., McGawley K. (2018). No individual or combined effects of caffeine and beetroot-juice supplementation during submaximal or maximal running. Appl. Physiol. Nutr. Metab..

[73] Wickham K.A., McCarthy D.G., Pereira J.M., Cervone D.T., Verdijk L.B., Van Loon L.J.C., Power G.A., Spriet L.L. (2019). No effect of beetroot juice supplementation on exercise economy and performance in recreationally active females despite increased torque production. Physiol. Rep..

[74] Wilkerson D.P., Hayward G.M., Bailey S.J., Vanhatalo A., Blackwell J.R., Jones A.M. (2012). Influence of acute dietary nitrate supplementation on 50 mile time trial performance in well-trained cyclists. Graefe’s Arch. Clin. Exp. Ophthalmol..

[75] Domínguez R., Cuenca E., Maté-Muñoz J.L., Garca-Fernández P., Paya N.S., Lozano-Estevan M.D.C., Veiga-Herreros P., Garnacho-Castaño M.V. (2017). Effects of Beetroot Juice Supplementation on Cardiorespiratory Endurance in Athletes. A Systematic Review. Nutrition.

[76] McMahon N.F., Leveritt M., Pavey T. (2016). The Effect of Dietary Nitrate Supplementation on Endurance Exercise Performance in Healthy Adults: A Systematic Review and Meta-Analysis. Sports Med..

[77] Van De Walle G.P., Vukovich M.D. (2018). The Effect of Nitrate Supplementation on Exercise Tolerance and Performance. J. Strength Cond. Res..

[78] Domínguez R., Maté-Muñoz J.L., Cuenca E., Garca-Fernández P., Ordoñez F.M., Estevan M.C.L., Veiga-Herreros P., Da Silva S.F., Garnacho-Castaño M.V. (2018). Effects of beetroot juice supplementation on intermittent high-intensity exercise efforts. J. Int. Soc. Sports Nutr..

[79] Spiegelhalder B., Eisenbrand G., Preussmann R. (1976). Influence of dietary nitrate on nitrite content of human saliva: Possible relevance to in vivo formation of N-nitroso compounds. Food Cosmet. Toxicol..

[80] Cosby K., Partovi K.S., Crawford J.H., Patel R.P., Reiter C.D., Martyr S., Yang B.K., Waclawiw M.A., Zalos G., Xu X. (2003). Nitrite reduction to nitric oxide by deoxyhemoglobin vasodilates the human circulation. Nat. Med..

[81] Kozlov A.V., Dietrich B., Nohl H. (2003). Various intracellular compartments cooperate in the release of nitric oxide from glycerol trinitrate in liver. Br. J. Pharmacol..

[82] Li H., Cui H., Kundu T.K., Alzawahra W., Zweier J.L. (2008). Nitric Oxide Production from Nitrite Occurs Primarily in Tissues Not in the Blood. J. Biol. Chem..

[83] Shiva S., Huang Z., Grubina R., Sun J., Ringwood L.A., MacArthur P.H., Xu X., Murphy E., Darley-Usmar V., Gladwin M.T. (2007). Deoxymyoglobin is a nitrite reductase that generates nitric oxide and regulates mitochondrial respiration. Circ. Res..

[84] Zhang Z., Naughton D.P., Blake D.R., Benjamin N., Stevens C.R., Winyard P.G., Symons M.C., Harrison R. (1997). Human xanthine oxidase converts nitrite ions into nitric oxide (NO). Biochem. Soc. Trans..

[85] Castello P.R., David P.S., McClure T., Crook Z.R., Poyton R.O. (2006). Mitochondrial cytochrome oxidase produces nitric oxide under hypoxic conditions: Implications for oxygen sensing and hypoxic signaling in eukaryotes. Cell Metab..

[86] Modin A., Björne H., Herulf M., Alving K., Weitzberg E., Lundberg J. (2001). Nitrite-derived nitric oxide: A possible mediator of ‘acidic-metabolic’ vasodilation. Acta Physiol. Scand..

[87] Richardson R.S., Noyszewski E.A., Kendrick K.F., Leigh J.S., Wagner P.D. (1995). Myoglobin O2 desaturation during exercise. Evidence of limited O2 transport. J. Clin. Investig..

[88] Gilliard C.N., Lam J.K., Cassel K.S., Park J.W., Schechter A.N., Piknova B. (2018). Effect of dietary nitrate levels on nitrate fluxes in rat skeletal muscle and liver. Nitric Oxide.

[89] Piknova B., Park J.W., Swanson K.M., Dey S., Noguchi C.T., Schechter A.N. (2015). Skeletal muscle as an endogenous nitrate reservoir. Nitric Oxide.

[90] Piknova B., Park J.W., Lam K.K.J., Schechter A.N. (2016). Nitrate as a source of nitrite and nitric oxide during exercise hyperemia in rat skeletal muscle. Nitric Oxide.

[91] Wylie L.J., Park J.W., Vanhatalo A., Kadach S., Black M.I., Stoyanov Z., Schechter A.N., Jones A.M., Piknova B. (2019). Human skeletal muscle nitrate store: Influence of dietary nitrate supplementation and exercise. J. Physiol..

[92] Webb A.J., Patel N., Loukogeorgakis S., Okorie M., Aboud Z., Misra S., Rashid R., Miall P., Deanfield J., Benjamin N. (2008). Acute blood pressure lowering, vasoprotective, and antiplatelet properties of dietary nitrate via bioconversion to nitrite. Hypertension.

[93] Dreißigacker U., Wendt M., Wittke T.-C., Tsikas D., Maassen N. (2010). Positive correlation between plasma nitrite and performance during high-intensive exercise but not oxidative stress in healthy men. Nitric Oxide.

[94] Fulford J., Winyard P.G., Vanhatalo A., Bailey S.J., Blackwell J.R., Jones A.M. (2013). Influence of dietary nitrate supplementation on human skeletal muscle metabolism and force production during maximum voluntary contractions. Pflüg. Arch..

[95] Haider G., Folland J.P. (2014). Nitrate Supplementation Enhances the Contractile Properties of Human Skeletal Muscle. Med. Sci. Sports Exerc..

[96] Whitfield J., Gamu D., Heigenhauser G.J.F., Van Loon L., Spriet L.L., Tupling A.R., Holloway G.P. (2017). Beetroot Juice Increases Human Muscle Force without Changing Ca2+-Handling Proteins. Med. Sci. Sports Exerc..

[97] Coggan A.R., Leibowitz J.L., Kadkhodayan A., Thomas D.P., Ramamurthy S., Spearie C.A., Waller S., Farmer M., Peterson L.R. (2015). Effect of acute dietary nitrate intake on maximal knee extensor speed and power in healthy men and women. Nitric Oxide.

[98] Hernández A., Schiffer T.A., Ivarsson N., Cheng A.J., Bruton J.D., Lundberg J.O., Weitzberg I., Westerblad H. (2012). Dietary nitrate increases tetanic [Ca2+]i and contractile force in mouse fast-twitch muscle. J. Physiol..

[99] Morton R., Sonne M.W., Zuniga A.F., Mohammad I., Jones A., McGlory C., Keir P.J., Potvin J.R., Phillips S.M. (2019). Muscle fibre activation is unaffected by load and repetition duration when resistance exercise is performed to task failure. J. Physiol..

[100] Bickel C.S., Gregory C.M., Dean J.C. (2011). Motor unit recruitment during neuromuscular electrical stimulation: A critical appraisal. Graefe’s Arch. Clin. Exp. Ophthalmol..

[101] Henneman E. (1957). Relation between Size of Neurons and Their Susceptibility to Discharge. Science.

[102] Allen D.G., Lamb G.D., Westerblad H. (2008). Skeletal Muscle Fatigue: Cellular Mechanisms. Physiol. Rev..

[103] Tillin N.A., Moudy S., Nourse K.M., Tyler C.J. (2018). Nitrate supplement benefits contractile forces in fatigued but not unfatigued muscle. Med. Sci. Sports Exerc..

[104] Bogdanis G.C., Nevill M.E., Boobis L.H., Lakomy H.K., Nevill A. (1995). Recovery of power output and muscle metabolites following 30 s of maximal sprint cycling in man. J. Physiol..

[105] Bogdanis G.C., Nevill M.E., Boobis L.H., Lakomy H.K. (1996). Contribution of phosphocreatine and aerobic metabolism to energy supply during repeated sprint exercise. J. Appl. Physiol..

[106] Gaitanos G.C., Williams C., Boobis L.H., Brooks S. (1993). Human muscle metabolism during intermittent maximal exercise. J. Appl. Physiol..

[107] Trump M.E., Heigenhauser G.J., Putman C.T., Spriet L.L. (1996). Importance of muscle phosphocreatine during intermittent maximal cycling. J. Appl. Physiol..

[108] Vanhatalo A., Fulford J., Bailey S.J., Blackwell J.R., Winyard P.G., Jones A.M. (2011). Dietary nitrate reduces muscle metabolic perturbation and improves exercise tolerance in hypoxia. J. Physiol..

[109] Ferguson S.K., Hirai D.M., Copp S.W., Holdsworth C.T., Allen J., Jones A.M., Musch T.I., Poole D.C. (2012). Impact of dietary nitrate supplementation via beetroot juice on exercising muscle vascular control in rats. J. Physiol..

[110] Shamseer L., Moher D., Clarke M., Ghersi D., Liberati A., Petticrew M., Shekelle P., Stewart L.A. (2015). The PRISMA-P Group Preferred reporting items for systematic review and meta-analysis protocols (PRISMA-P) 2015: Elaboration and explanation. BMJ.

[111] Maher C.G., Sherrington C., Herbert R.D., Moseley A.M., Elkins M.R. (2003). Reliability of the PEDro Scale for Rating Quality of Randomized Controlled Trials. Phys. Ther..

[112] Brown W.R., Brunnhuber K., Chalkidou K., Chalmers I., Clarke M., Fenton M., Forbes C., Glanville J., Hicks N.J., Moody J. (2006). How to formulate research recommendations. BMJ.

[113] Williams T.D., Martin M.P., Mintz J.A., Rogers R.R., Ballmann C.G. (2020). Effect of Acute Beetroot Juice Supplementation on Bench Press Power, Velocity, and Repetition Volume. J. Strength Cond. Res..

[114] Ranchal-Sanchez A., Diaz-Bernier V.M., De La Florida-Villagran C.A., Llorente-Cantarero F.J., Campos-Perez J., Jurado-Castro J.M. (2020). Acute Effects of Beetroot Juice Supplements on Resistance Training: A Randomized Double-Blind Crossover. Nutrients.

[115] Flanagan S.D., Looney D.P., Miller M.J.S., Dupont W.H., Pryor L., Creighton B.C., Sterczala A.J., Szivak T.K., Hooper D.R., Maresh C.M. (2016). The Effects of Nitrate-Rich Supplementation on Neuromuscular Efficiency during Heavy Resistance Exercise. J. Am. Coll. Nutr..

[116] Mosher S.L., Sparks S.A., Williams E.L., Bentley D., McNaughton L.R. (2016). Ingestion of a Nitric Oxide Enhancing Supplement Improves Resistance Exercise Performance. J. Strength Cond. Res..

[117] Del Coso J., Salinero J.J., González-Millán C., Abián-Vicén J., Pérez-González B. (2012). Dose response effects of a caffeine-containing energy drink on muscle performance: A repeated measures design. J. Int. Soc. Sports Nutr..

[118] Mora-Rodriguez R., Pallarés J.G., López-Gullón J.M., López-Samanes Á., Fernández-Elías V.E., Ortega J.F. (2015). Improvements on neuromuscular performance with caffeine ingestion depend on the time-of-day. J. Sci. Med. Sport.

[119] Pallarés J.G., Fernández-Elías V.E., Ortega J.F., Muñoz G., Muñoz-Guerra J., Mora-Rodriguez R. (2013). Neuromuscular Responses to Incremental Caffeine Doses. Med. Sci. Sports Exerc..

[120] Maté-Muñoz J.L., Lougedo J.H., Garnacho-Castaño M.V., Veiga-Herreros P., Estevan M.D.C.L., Garca-Fernández P., De Jesús F., Guodemar-Pérez J., Juan A.F.S., Domínguez R. (2018). Effects of β-alanine supplementation during a 5-week strength training program: A randomized, controlled study. J. Int. Soc. Sports Nutr..

[121] Venier S., Grgic J., Mikulic P., Veiner S. (2019). Acute Enhancement of Jump Performance, Muscle Strength, and Power in Resistance-Trained Men After Consumption of Caffeinated Chewing Gum. Int. J. Sports Physiol. Perform..

[122] Romero-Moraleda B., Del Coso J., Gutiérrez-Hellín J., Lara B. (2019). The Effect of Caffeine on the Velocity of Half-Squat Exercise during the Menstrual Cycle: A Randomized Controlled Trial. Nutrients.

[123] Coggan A.R., Broadstreet S.R., Mikhalkova D., Bole I., Leibowitz J.L., Kadkhodayan A., Park S., Thomas D.P., Thies D., Peterson L.R. (2018). Dietary nitrate-induced increases in human muscle power: High versus low responders. Physiol. Rep..

[124] Jones A.M., Ferguson S.K., Bailey S.J., Vanhatalo A., Poole D.C. (2016). Fiber Type-Specific Effects of Dietary Nitrate. Exerc. Sport Sci. Rev..

[125] Ørtenblad N., Nielsen J., Boushel R., Söderlund K., Saltin B., Holmberg H.-C. (2018). The Muscle Fiber Profiles, Mitochondrial Content, and Enzyme Activities of the Exceptionally Well-Trained Arm and Leg Muscles of Elite Cross-Country Skiers. Front. Physiol..

[126] Andersen J.L., Aagaard P. (2000). Myosin heavy chain IIX overshoot in human skeletal muscle. Muscle Nerve.

[127] Andersen L.L., Andersen J.L., Zebis M.K., Aagaard P. (2010). Early and late rate of force development: Differential adaptive responses to resistance training?. Scand. J. Med. Sci. Sports.

[128] Coggan A.R., Hoffman R.L., Gray D.A., Moorthi R.N., Thomas D.P., Leibowitz J.L., Thies D., Peterson L.R. (2019). A Single Dose of Dietary Nitrate Increases Maximal Knee Extensor Angular Velocity and Power in Healthy Older Men and Women. J. Gerontol. Ser. A Biol. Sci. Med. Sci..

[129] Cuenca E., Jodra P., Pérez-López A., Rodríguez L.G.G., Da Silva S.F., Veiga-Herreros P., Domínguez R. (2018). Effects of Beetroot Juice Supplementation on Performance and Fatigue in a 30-s All-Out Sprint Exercise: A Randomized, Double-Blind Cross-Over Study. Nutrients.

[130] Domínguez R., Garnacho-Castaño M.V., Cuenca E., García-Fernández P., Muñoz-González A., De-Jesús-Franco F., Estevan M.C.L., Da Silva S.F., Veiga-Herreros P., Maté-Muñoz J.L. (2017). Effects of Beetroot Juice Supplementation on a 30-s High-Intensity Inertial Cycle Ergometer Test. Nutrients.

[131] Jodra P., Domínguez R., Sánchez-Oliver A.J., Veiga-Herreros P., Bailey S.J. (2020). Effect of Beetroot Juice Supplementation on Mood, Perceived Exertion, and Performance During a 30-Second Wingate Test. Int. J. Sports Physiol. Perform..

[132] Kramer S.J., Baur D.A., Spicer M.T., Vukovich M.D., Ormsbee M. (2016). The effect of six days of dietary nitrate supplementation on performance in trained CrossFit athletes. J. Int. Soc. Sports Nutr..

[133] Wylie L.J., Bailey S.J., Kelly J., Blackwell J.R., Vanhatalo A., Jones A.M. (2015). Influence of beetroot juice supplementation on intermittent exercise performance. Graefe’s Arch. Clin. Exp. Ophthalmol..

[134] Andrade F.H., Reid M.B., Allen D.G., Westerblad H. (1998). Effect of hydrogen peroxide and dithiothreitol on contractile function of single skeletal muscle fibres from the mouse. J. Physiol..

[135] Bailey S.J., Gandra P.G., Jones A.M., Hogan M.C., Nogueira L. (2019). Incubation with sodium nitrite attenuates fatigue development in intact single mouse fibres at physiological. J. Physiol..

[136] Nyakayiru J., Kouw I.W., Cermak N.M., Senden J.M., Van Loon L.J.C., Verdijk L.B. (2017). Sodium nitrate ingestion increases skeletal muscle nitrate content in humans. J. Appl. Physiol..

[137] Stamler J.S., Meissner G. (2001). Physiology of nitric oxide in skeletal muscle. Physiol. Rev..

[138] Stamler J.S. (1994). Redox signaling: Nitrosylation and related target interactions of nitric oxide. Cell.

[139] Evangelista A.M., Rao V.S., Filo A.R., Marozkina N.V., Doctor A., Jones D.R., Gaston B., Guilford W. (2010). Direct Regulation of Striated Muscle Myosins by Nitric Oxide and Endogenous Nitrosothiols. PLoS ONE.

[140] Nogueira L., Figueiredo-Freitas C., Casimiro-Lopes G., Magdesian M.H., Assreuy J., Sorenson M.M. (2009). Myosin is reversibly inhibited by S-nitrosylation. Biochem. J..

[141] Dutka T.L., Mollica J.P., Lamboley C.R., Weerakkody V.C., Greening D.W., Posterino G.S., Murphy R.M., Lamb G.D. (2017). S-nitrosylation and S-glutathionylation of Cys134 on troponin I have opposing competitive actions on Ca2+ sensitivity in rat fast-twitch muscle fibers. Am. J. Physiol. Physiol..

[142] Ishii T., Sunami O., Saitoh N., Nishio H., Takeuchi T., Hata F. (1998). Inhibition of skeletal muscle sarcoplasmic reticulum Ca2+-ATPase by nitric oxide. FEBS Lett..

[143] Eu J.P., Sun J., Xu L., Stamler J.S., Meissner G. (2000). The Skeletal Muscle Calcium Release Channel. Cell.

[144] Stoyanovsky D., Murphy T., Anno P.R., Kim Y.-M., Salama G. (1997). Nitric oxide activates skeletal and cardiac ryanodine receptors. Cell Calcium.

[145] Gould N., Doulias P.-T., Tenopoulou M., Raju K., Ischiropoulos H. (2013). Regulation of Protein Function and Signaling by Reversible Cysteine S-Nitrosylation*. J. Biol. Chem..

[146] Takeshima H., Nishimura S., Matsumoto T., Ishida H., Kangawa K., Minamino N., Matsuo H., Ueda M., Hanaoka M., Hirose T. (1989). Primary structure and expression from complementary DNA of skeletal muscle ryanodine receptor. Nature.

[147] Thompson C., Vanhatalo A., Kadach S., Wylie L.J., Fulford J., Ferguson S.K., Blackwell J.R., Bailey S.J., Jones A.M. (2018). Discrete physiological effects of beetroot juice and potassium nitrate supplementation following 4-wk sprint interval training. J. Appl. Physiol..

[148] Coffey V.G., Hawley J.A. (2016). Concurrent exercise training: Do opposites distract?. J. Physiol..

[149] Fyfe J.J., Bishop D., Stepto N.K. (2014). Interference between Concurrent Resistance and Endurance Exercise: Molecular Bases and the Role of Individual Training Variables. Sports Med..

[150] Pride C.K., Mo L., Quesnelle K.M., Dagda R.K., Murillo D., Geary L., Corey C., Portella R., Zharikov S., Croix C.S. (2013). Nitrite activates protein kinase A in normoxia to mediate mitochondrial fusion and tolerance to ischaemia/reperfusion. Cardiovasc. Res..

